# Sub-typing of renal cell tumours; contribution of ancillary techniques

**DOI:** 10.1186/1746-1596-4-21

**Published:** 2009-06-28

**Authors:** Dinesh Pradhan, Nandita Kakkar, Amanjit Bal, Shrawan Kumar Singh, Kusum Joshi

**Affiliations:** 1 Department of Histopathology, Post Graduate Institute of Medical Sciences & Research, (PGIMER), Chandigarh, India; 2 Advanced Urology Centre, Post Graduate Institute of Medical Sciences & Research, (PGIMER), Chandigarh, India

## Abstract

**Background:**

Adult renal epithelial neoplasms are a heterogeneous group with varying prognosis and outcome requiring sub-classification.

**Methods:**

Cases of renal cell carcinoma (RCC) in a 10 years period were analyzed with regard to the clinical features and histology. Sections were reviewed by four pathologists and the discordant cases were resolved with the help of Hale's colloidal iron stain, vimentin, CK 7, and vinculin immunostains and electron microscopy.

**Results:**

Amongst the total of 278 cases, clear cell renal cell carcinoma was the commonest tumor with 74.8% cases, followed by papillary RCC 12.2%, chromophobe RCC 7.9%, oncocytoma 1.8%, and one case of collecting duct RCC. Eight cases were of sarcomatoid renal cell carcinoma. In 28/278 cases, diagnoses varied amongst the four pathologists and the discordance was resolved by Hale's colloidal iron stain, CK7 immunostain and electron microscopy. Vimentin and vinculin did not contribute much in differentiating subtypes of renal cell carcinomas. Relative incidence of sub-types of RCCs was compared with other series

**Conclusion:**

To accurately subclassify renal cell carcinomas, simple ancillary techniques would possibly resolve all difficult cases. The relative incidence of sub-types of renal cell carcinoma is relatively consistent the world over. However, in India, RCCs afflict the patients two decades earlier.

## Background

Renal cell carcinoma (RCC), accounts for 2–3% of all new cancers diagnosed and 85% of all primary renal neoplasms in adults[1]. Adult renal epithelial neoplasms are a heterogeneous group comprised of subtypes that have distinct gross, histologic, ultrastructural, and immunohistochemical features. These morphologic distinctions are amply supported by unique cytogenetic and chromosomal aberrations for many of the subtypes[2,3]. Thus classification of renal cell carcinoma is important from the treatment and prognosis point of view as well as for understanding its histogenesis, molecular and cytogenetic behaviour for further improvement in its management approach.

Keeping this in view, many classification systems have been made till date. Mainz classification[4] and subsequently AFIP classification[5] sub-classified these tumours purely based on morphological grounds. However, the first classification based on molecular and cytogenetic studies and on the cell of origin of each entity came into being in 1997 as the Heidelberg classification[6]. Subsequently, WHO classified renal cell tumors into clear cell RCC, multilocular clear cell RCC, papillary RCC, chromophobe RCC, carcinoma of the collecting ducts of Bellini, renal medullary carcinoma, Xp11 translocation carcinoma, carcinoma associated with neuroblastoma, mucinous tubular and spindle cell carcinoma, renal cell carcinoma-unclassified, papillary adenoma and oncocytoma[7].

Whilst a majority of renal cell carcinomas seen in routine practice of surgical pathology, are easy to diagnose based on haematoxylin and eosin preparation alone, there is insufficient information on how to deal with the differential diagnoses regarding subtypes of RCC. The present study aims at classifying adult renal tumors based on the cell of origin, by histomorphology, immunohistochemistry, and ultrastructural studies.

## Methods

The material of this study was derived from cases of renal cell carcinoma received in the Department of Histopathology, Post Graduate Institute of Medical Education and Research PGIMER, Chandigarh from July, 1997 to June, 2007 (ten years period).

Patients aged more than 16 years were included in the study. All the cases were routinely fixed in 10% buffered formalin. 4 μ sections were cut and haematoxylin and eosin staining was performed in routine using the standardized methods. Haematoxylin and eosin stained sections were reviewed by independent pathologists in a blinded manner and the diagnosis of the four pathologists were compared. All cases with discordant diagnosis or in which a definitive diagnosis on the basis of morphology was not possible were taken up for further study which included histochemical, immunohistochemical, and ultrastructural analysis.

### Hale's colloidal iron stain

Cases in which chromophobe cell carcinoma was kept as possibility by any of the three observers were taken up for Hale's colloidal iron stain. The method of Hale's colloidal iron stain used was a modification published by Tickoo et al[8].

### Immunohistochemistry

All the cases with discordant diagnoses were taken up for immunohistochemistry; vimentin (Dako Cytomation, 1:50 dilution), Cytokeratin 7(Dako Cytomation, 1:50 dilution), and vinculin (Santa Cruz Biotechnology, 1:40 dilution) according to the differential diagnosis kept in a given case and with a minimum of 5 cases in each subgroup and including all 5 oncocytomas.

Immunostaining was carried out on 4 μ paraffin sections after antigen retrieval using pressure cooker method. The sections were incubated with appropriately diluted primary antibody, washed in PBS (3 × 5 min) and then incubated with peroxidase conjugated secondary antibody. Color reaction was developed by DAB and counterstained by hematoxylin. Appropriate positive and negative controls were taken. Immunoreactivity was evaluated by taking into account the percentage positivity of tumor cells. Positivity was taken as a brown reaction product staining the cytoplasm. The cells were scored as negative or positive and the percentage of positive tumor cells were recorded, which ranged from 0–100%. The percentage positivity was graded from 1+ to 3+ as follows:-

5–25%   -- 1+

25–75%   -- 2+

>75%   -- 3+

### Electron Microscopy

Ultrastructural analysis of selected cases was done to confirm the diagnosis, when required. The tissue for electron microscopy was taken from formalin fixed tissue of all cases of oncocytoma and representative cases from discordant groups. Tissue blocks were fixed in 3% buffered glutaraldehyde and processed for electron microscopy by routine methods. The grids were examined under Zeiss 906 electron microscope, and representative photographs were taken under suitable magnification.

## Results

A total of 278 cases of renal cell carcinoma were included in this study. Age of the patients ranged from 16–78 years, the average being 52 years (mean = 51.45). The peak incidence was in the fourth and fifth decades and the male to female ratio of 2.3:1. Out of these 278 renal carcinomas, concordant diagnoses were obtained in 250 cases whereas in the remaining 28 cases, diagnosis varied amongst the four pathologists. The discordance was resolved by histochemistry, immunohistochemistry and electron microscopy as detailed in Table 1. Table 2 shows incidence of sub-types of RCCs amongst the total of 278 cases.

**Table 1:** Detailed Description of Discordant Cases (N = 28)

**S. No**.	**Diagnosis**	**Hale's stain**	**Vimentin**	**CK 7**	**Vinculin**	**EM**	**Final Diagnosis**
1	CCRCC vs papillary		-	-	-		Papillary RCC
2	CCRCC vs Chromophobe	-	+	-	-		Conventional RCC
3	Papillary RCC vs CCRCC		-	-	-		Conventional RCC
4	Chromophobe RCC vs CCRCC	+	+	3+, m	-	PNV	Chromophobe RCC
5	Papillary RCC vs CCRCC		3+	+	-		Papillary RCC
6	Collecting duct RCC vs Papillary		2+	3+	-		Papillary RCC
7	CCRCC vs papillary		+	2+	-		Papillary RCC
8	ACC vs CCRCC		3+	-	-		Conventional RCC
9	Chromophobe RCC vs CRCC	+	-	2+, m	-		Chromophobe RCC
10	CCRCC vs Papillary		+	-	-		Conventional RCC
11	Collecting duct RCC vs CCRCC		2+	2+	2+		Collecting duct RCC
12	CCRCC vs oncocytoma		-	-	-		Conventional RCC
13	Chromophobe vs CCRCC	-	+	-	-		Conventional RCC
14	Chromophobe vs CCRCC	+	-	2+, m	-	PNV	Chromophobe RCC
15	Chromophobe vs CCRCC	-	-	-	-		Conventional RCC
16	CCRCC vs papillary		-	+	-		Papillary RCC
17	CCRCC vs Sarcomatoid		-	-	-		Sarcomatoid RCC
18	Papillary RCC vs CCRCC		+	2+	-		Papillary RCC
19	Chromophobe vs CCRCC	+	-	2+, m	-		Chromophobe RCC
20	Chromophobe RCC vs CCRCC	-	-	-	-	Glycogen	Conventional RCC
21	Chromophobe vs CCRCC	-	2+	-	-		Conventional RCC
22	CCRCC vs chromophobe	-	3+	3+	-		Papillary RCC
23	Chromophobe vs oncocytoma		-	3+	-	PNV	Chromophobe RCC
24	CC RCC vs papillary		2+	3+	-	No PNV	Papillary RCC
25	Chromophobe vs CCRCC	+	2+	+	-		Chromophobe RCC
26	CCRCC vs Chromophobe	+	-	3+, m	-		Chromophobe RCC
27	CCRCC vs papillary		-	-	-		Conventional RCC
28	Oncocytoma vs ACC vs CCRCC		-	-	-		Conventional RCC

CCRCC – Conventinal/clear cell RCC, ACC – adrenocortical carcinoma, m – membranous accentuation, PNV-Perinuclear vesicles

**Table 2:** Sub-types of Renal Cell Carcinomas amongst total cases (N = 278)

**SUBTYPES OF RCC**	**INCIDENCE (%)** **Concordant cases** **(n = 250)**	**INCIDENCE (%)** **Total Cases** **(n = 278)**
**Conventional or clear cell RCC (CCRCC)**	197(78.8)	208(74.8)
**Papillary RCC (PRCC)**	26(10.4)	34(12.2)
**Chromophobe RCC (CRCC)**	15(6)	22(7.9)
**Renal oncocytoma (RO)**	5(2)	5(1.8)
**Collecting duct RCC (CDRCC)**	0(0)	1 (0.4)
**Sarcomatoid RCC (SRCC)**	7(2.8)	8(2.9)

***Clear Cell RCC ***was the most common kidney tumor accounting for 74.8% (208/278) of all adult renal tumors. They exhibited a male preponderance and mean age at presentation of 56.2 years. Clear cell RCC were mostly solitary and bilaterality was noted in a single case. Fuhrman grading revealed that 85% of clear cell renal cell carcinomas were grade 1 and 2, and less than 5% were grade 4. Multicystic clear cell renal cell carcinoma was noted in 5 cases, 4 of which were nuclear grade 1 and one case was nuclear grade 2. Cytoplasmic inclusions were noted in 78 cases. Well formed psammoma bodies and fibro-calcific bodies were seen in 2 cases each; however calcification, cholesterol clefts, hemosiderin pigment and hyaline globules were seen in many cases. Areas with rhabdoid differentiation were noted in 2 cases while granulomatous inflammation was noted in the adjoining lymph node in another 2 cases. Sarcomatoid change was seen in 5 cases. One case revealed membranous glomerulonephritis in the adjoining renal parenchyma. Invasion of peri-renal fat and extension into the renal vein with thrombosis was noted in two cases.

***Papillary (or chromophilic) renal cell carcinomas ***comprised 12.2% (n = 34) of cases. The mean age at presentation was 52.4 years and the sex ratio was M: F = 2.1:1. Based upon morphology two types of PRCC were categorized; Type 1 tumours (27/34 cases) had papillae covered by small cells with scanty basophilic cytoplasm, arranged in a single layer on the papillary basement membrane and Type 2 tumours (7/34 cases) had cells of higher nuclear grade with eosinophillic cytoplasm and pseudostratified nuclei on papillary cores. Out of the 34 cases of papillary RCC, 6 (17.6%) cases were Fuhrman's nuclear grade 1, 15 (44.1%) cases were grade 2, 11 (32.4%) cases were grade 3 and 2

(5.9%) cases were grade 4. Sarcomatoid dedifferentiation was seen in only 1 case. Four cases showed psammoma bodies and 1 case showed fibro-siderotic nodules. Invasion of peri-renal fat and extension into the renal vein with thrombosis was noted in one case of PRCC. Another 1 case of PRCC showed lymph node metastasis.

***Chromophobe renal cell carcinomas ***comprised 7.9% (n = 22) of all renal cell carcinomas. The mean age at presentation was 46.8 years and there was a slight female preponderance. The eosinophilic variant of chromophobe carcinoma was found in 7 cases and was purely composed of intensely eosinophilic cells with prominent cell membranes. Four cases (18.2%) were Fuhrman's nuclear grade 1, 11 (50%) cases were grade 2, 6 (27.3%) cases were grade 3 and 1 (4.5%) case was grade 4. Well formed psammoma bodies were seen in 2 cases, however, calcification, cholesterol clefts, hemosiderin pigment and hyaline globules were seen in many cases. Sarcomatoid transformation was noted in 2 cases.

***Renal oncocytoma ***comprised 1.8% (n = 5) of all renal cell carcinomas. The mean age at presentation was 48 years with a slight female preponderance. The cells were round-to polygonal with densely granular eosinophilic cytoplasm.

### Others

Only one case of collecting duct carcinoma in a 70 year male was seen. The cells of collecting duct carcinoma displayed high nuclear grade (Fuhrman 4). Sarcomatoid RCC was noted in 2.9%(n = 8) of all adult renal cell carcinomas. The mean age at presentation was 66.2 years with a male preponderance. As the sarcomatoid element overshadowed the original antecedent carcinoma to the extent that it could not be recognized, so it was kept under Sarcomatoid RCC or unclassified category (according to the recent WHO).

### Discordant cases (Table 1)

**Figure 1:**
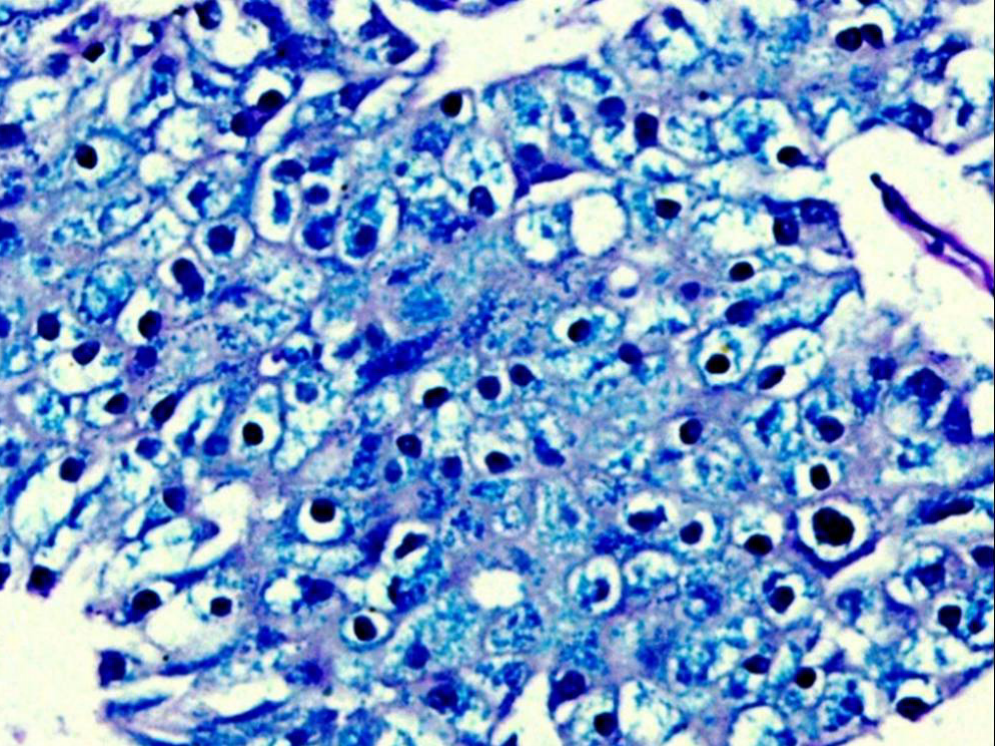
**Photomicrograph of Hale's colloidal iron stain showing deep blue granular cytoplasmic positivity in chromophobe RCC (HCI with hematoxylin counterstain, ×200)**.

Most (12/28 cases) of the discordance was between clear cell RCC and chromophobe RCC. This was solved by Hale's colloidal iron stain which yielded a diffuse blue granular cytoplasmic staining in chromophobe renal cell carcinoma(Fig. 1) and pink periodic acid Schiff positivity in cytoplasm of clear cell renal cell carcinoma cells. Other subtypes of RCC were negative. This stain was very helpful and statistically significant (p < 0.0001) in differentiating chromophobe renal cell carcinoma from other subtypes of RCC. CK7 was also positive in all cases of chromophobe RCC and negative in clear cell RCC. Next group of discordance was between clear cell RCC, with papillary areas and papillary RCC (10/28) which was resolved by CK7 immunostain which was positive in most of the cases (90%) of papillary RCC and was negative in all cases of clear cell RCC. Third group of discordance was between eosinophilic variant of chromophobe RCC and oncocytoma. This discordance was resolved by CK7 immunostain which was positive in all cases of chromophobe RCC and was negative in oncocytoma. Electron microscopy was of diagnostic importance in oncocytomas by showing cytoplasm packed with mitochondria to the exclusion of other organelles.

### Immunohistochemistry

***Vimentin ***was positive in 53.9% (7/13) cases of clear cell renal cell carcinoma and 80% (8/10) cases of papillary renal cell carcinoma. It was positive in 2 (20%) cases of chromophobe RCC. One case of collecting duct RCC showed focal positivity in stromal cells. Oncocytomas showed negative vimentin staining (0/5).

**Figure 2:**
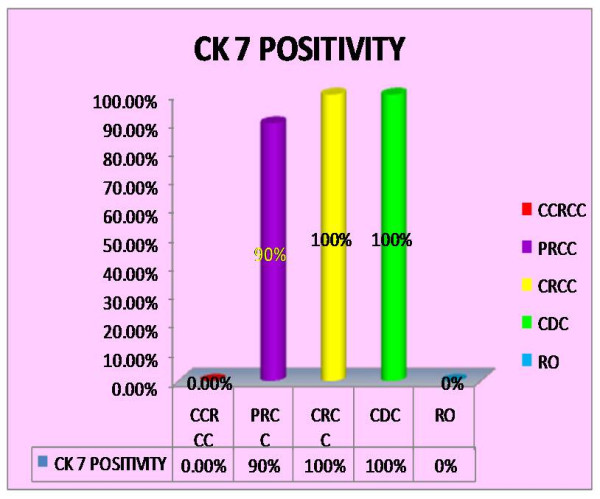
**Bar diagram showing CK7 positivity in RCC(CCRCC-Clear cell renal cell carcinoma; PRCC-Papillary renal cell carcinoma; CRCC-Chromophobe renal cell carcinoma; CDC-Collecting duct carcinoma; RO-Renal Oncocytoma)**.

**Figure 3:**
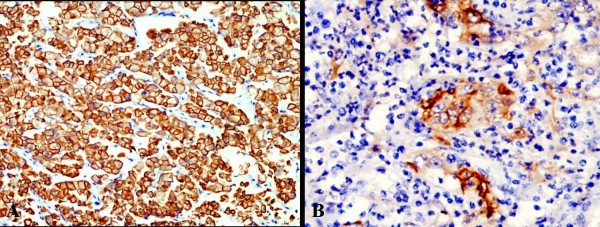
**Immunohistochemical staining using CK7 antibody showing strong and diffuse membranous positivity in chromophobe renal cell carcinoma(A) and collecting duct carcinoma(B) (CK7, ×200)**.

***CK 7 ***(Fig. 2 and 3): was positive in 90%(9/10) cases of papillary renal cell carcinoma (p < 0.001) and was more frequently observed in type 1 (100%) than type 2 (75%) tumours. All the cases of chromophobe renal cell carcinoma (10/10) and collecting duct carcinoma (1/1) were positive. It was negative in all 20 cases of clear cell renal cell carcinoma and 5 cases of renal oncocytoma.

**Figure 4:**
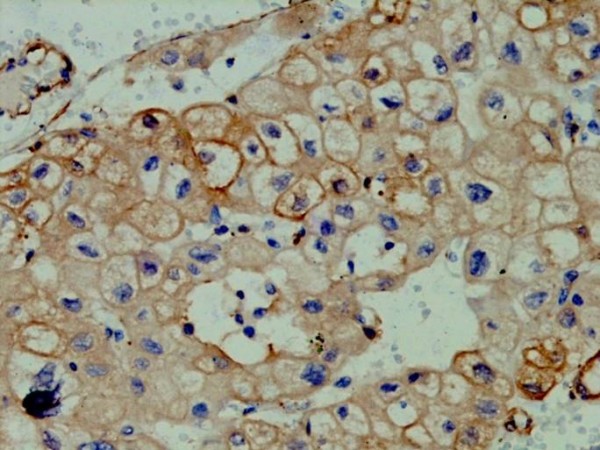
**Photomicrograph showing weak but distinct vinculin positivity on the cell membrane of chromophobe RCC(Vinculin, ×200)**.

***Vinculin ***(Fig. 4) was weakly but distinctly positive on the cell membrane of only 2/15 cases of chromophobe RCC and 1 case of collecting duct RCC. It was negative in all cases of clear cell renal cell carcinoma (0/13), papillary renal cell carcinoma (0/12) and renal oncocytoma (0/5). It was not found to be useful in resolving the differential diagnosis because of its low sensitivity in cases of chromophobe RCC.

### Electron Microscopy

**Figure 5:**
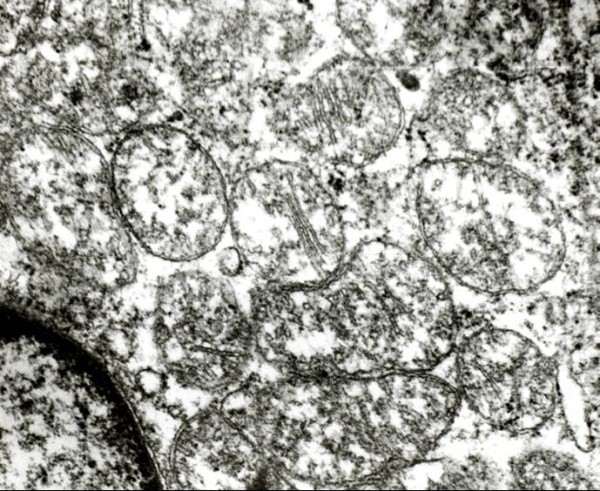
**Electron microscopy showing numerous mitochondria packing the cytoplasm in oncocytoma (Uranyl acetate and lead citrate, × 10,000)**.

Ultrastructural examination was done in renal oncocytoma(5 cases), chromophobe renal cell carcinoma (7 cases), and 2 cases each of eosinophilic variants of clear cell renal cell carcinoma and type 2 papillary RCC. Special attention was paid to mitochondria and microvesicles and interrelations thereof. The cytoplasm of all the oncocytomas were packed with abundant mitochondria (Fig. 5). Although abundant microvesicles were present in all the chromophobe renal cell carcinomas, but scant numbers of microvesicles were also present in renal oncocytomas and in the eosinophilic variant of clear cell renal cell carcinoma. The mitochondria in all three types of renal neoplasms studied differed in morphology, being predominantly uniform and round with prominent lamellar cristae packing the cytoplasm in renal oncocytoma. Ultrastructural examination of papillary RCC showed basal infoldings and increased mitochondria with glycogen granules.

## Discussion

Classification of renal cell carcinoma is important from the treatment and prognosis point of view as well as for understanding of histogenesis. The main objective of this study was to establish the degree of inter-observer variation in sub-classification of renal cell carcinomas and to find out means of subtyping renal cell carcinoma short of cytogenetic studies. In the present study, cases were seen by four independent pathologists in a blinded manner and their diagnoses were compared. Out of these 278 renal tumors, concordant diagnosis was obtained in 250 cases whereas in the remaining 28 cases, diagnoses varied amongst the four pathologists. This data emphasizes the fact that haematoxylin and eosin histology alone is sufficient to classify a majority of renal cell carcinoma. However help from ancillary techniques may be required in approximately 10% of the cases.

Amongst the 278 cases, clear cell RCC was the commonest tumor with 208 (74.8%) cases, followed by papillary RCC with 34(12.2%) cases, and chromophobe RCC with 22(7.9%) cases. There were only 5(1.8%) cases of renal oncocytoma, 1 (0.4%) case of collecting duct RCC, and 8 (2.9%) cases of RCC, unclassified. This is comparable with the world literature, both western and Asian literature where clear cell RCC is the most common adult renal tumor with an incidence of as high as 75% of all RCC followed by papillary RCC which comprise 7–14% of RCCs (Table 3)[6,9-15]. Indian study by Srivastava et al[16] also showed clear cell RCC to be the most common adult renal tumor with 114/178 (64.02%) cases (Table 3).

**Table 3:** Comparison of incidence of Sub-types of Renal Cell Carcinomas with Western and Asian studies

	**CCRCC**	**PRCC**	**CRCC**	**RO**	**CDC**	**UNCLASSIFIED**
**Present Study(India)**	208 (74.8%)	34(12.2%)	22(7.9%)	5(1.8%)	1(0.5%)	-
**Srivastva et al **[17]**(India)**	114 (64.0%)	48 (27.0%)	14 (7.86%)	-	2 (1.12%)	-
**Zou et al **[12]**(China)**	77(67.5%)	11(9.6%)	14(12.3%)	-	-	2(1.8%)
**Patard et al**[13]**(France)**	3564(87.7%)	396(9.7%)	103(2.5%)	-	-	-
**Kim et al**[14]**(Korea)**	686(86.3%)	58(7.3%)	49(6.16%)	-	2(0.25%)	-
**Gudbjartsson et al**[15]**(Iceland)**	558(88.7%)	53(8.4%)	13(2.1%)	-	-	-
**Kovacs et al **[6]**(Japan)**	80%	10%	5%	5%	-	-
**Amin et al**[16]**(USA)**	255(63%)	75(18.5%)	24(5.9%)	-	-	23(5.7%)

The peak incidence of RCC in this study was in the fourth and fifth decades, in contrast to other studies in the western world, where the majority of cases were in their sixth and seventh decades [9-11]. The data in the present study also showed that the mean age of patients with chromophobe RCC and renal oncocytoma were less than the other sub-types. Literature from the western world show two to three fold male predominance in renal tumors. The data in the present study also showed similar results with a sex ratio, M: F = 2.3:1. However, the sex ratio was altered in chromophobe RCC and renal Oncocytoma where a marginal female predominance was seen. This data also varied with the world literature which shows male predominance in oncocytoma and equal sex incidence in chromophobe RCC [9-11].

Fuhrman grading of most (85%) cases of clear cell renal cell carcinomas in our study was grade 1 and 2, and less than 5% was nuclear grade 4. This data, however varied from the study by Srivastava et al[16] who showed 14.6% grade 1 tumor, 38.2% grade 2 tumor, 35.4% grade 3 tumor and 11.8% grade 4 tumor. Sarcomatoid change was seen in 5 cases of clear cell renal cell carcinoma, and in one case of papillary renal cell carcinoma. This is consistent with literature which shows less than 5% clear cell renal cell carcinoma with sarcomatoid dedifferentiation[10].

Vimentin was positive in 53.9% cases of clear cell renal cell carcinoma, 80% cases of papillary renal cell carcinoma, focally positive in one case of chromophobe RCC and diffusely positive in another case of chromophobe RCC with sarcomatoid areas. One case of collecting duct RCC showed focal positivity in stromal cells, however, oncocytomas (0/5) showed negative vimentin staining. The result was comparable to the study by Dierick et al[17] who also found 53.3% positivity in cases of renal cell carcinoma. Similar findings have been reported by Waldherr et al[18] and by Holthofer et al[19]. However, vimentin immunostain did not prove to be useful in differential diagnosis of subtypes of renal cell carcinoma.

In this study, CK 7 was positive in 90%(9/10) cases of papillary renal cell carcinoma, 100% cases of chromophobe renal cell carcinoma, and the single case of collecting duct carcinoma. It was negative in all cases of clear cell renal cell carcinoma and renal oncocytoma. This result is comparable to the studies by Mathers et al[20] and Leroy et al[21] who showed positivity of CK7 in 100% cases of chromophobe renal cell carcinoma, and one case of clear cell renal cell carcinoma. Study by Yang et al[22] showed 87–100% positivity of CK7 in papillary renal cell carcinoma. Hence, according to present study, CK7 is a helpful immunostain in differentiating ambiguous cases, particularly chromophobe versus clear cell RCC and papillary RCC versus conventional RCC with a papillary pattern.

Vinculin was weakly positive in only 2 out of 15 cases of chromophobe RCC, and single case of collecting duct RCC and was negative in all cases of clear cell renal cell carcinoma, papillary renal cell carcinoma and renal oncocytoma. Hence in this study, the vinculin immunostain was not of much use in resolving difficult cases. However, study by Kuroda et al[23] found vinculin positivity in 21.5% of all RCC.

The tinctorial characteristics of different renal epithelial neoplasms appear to be dependent on cytoplasmic contents, including various organelles[24]. Differential diagnoses between eosinophilic variant of clear cell renal cell carcinoma, oxyphil variant of chromophobe renal cell carcinoma and renal oncocytoma was resolved by electron microscopy. The mitochondria in these three types of renal neoplasms differed in morphology, being predominantly uniform and round with predominantly lamellar cristae in renal oncocytoma, variable in shape and size with predominantly tubulocystic cristae in chromophobe renal cell carcinoma, and swollen and pleomorphic with rarefied matrix and attenuated cristae in the eosinophilic variant of conventional (clear cell) renal cell carcinoma.

## Conclusion

It can be concluded that incidence of different subtypes of renal tumours is similar to western and other Asian countries, however the age of presentation is one decade earlier than western population. In order to accurately subclassify renal cell carcinomas, the ancillary techniques required in a good surgical pathology set up consist of Hale's colloidal iron stain, immunostain CK7 and electron microscopy. With the help of these three ancillary techniques it would be possible to resolve all difficult cases that one may be faced with.

## Competing interests

The authors declare that they have no competing interests.

## Authors' contributions

DP and KJ participated in selecting cases, carrying out immunohistochemistry, interpretation of results, and writing of the manuscript. NK and AB participated in the histopathological diagnosis, and editing of the manuscript. SKS provided the clinical details of the patients. All authors read and approved the final manuscript.
